# Interferon-Alpha Triggers B Cell Effector 1 (Be1) Commitment

**DOI:** 10.1371/journal.pone.0019366

**Published:** 2011-04-29

**Authors:** Marie-Ghislaine de Goër de Herve, Deniz Durali, Bamory Dembele, Massimo Giuliani, Tu-Anh Tran, Bruno Azzarone, Pierre Eid, Marc Tardieu, Jean-François Delfraissy, Yassine Taoufik

**Affiliations:** 1 INSERM U-1012 and Université Paris-Sud, Bicêtre, France; 2 unité d'Immunologie Biologique, hôpital de Bicêtre, Bicêtre, France; 3 TUBITAK Marmara Research Center, Genetic Engineering and Biotechnology Institute (GEBI), Vaccine Technology Laboratory, Gebze-Kocaeli, Turkey; 4 INSERM U-542, Villejuif, France; New York University, United States of America

## Abstract

B-cells can contribute to the pathogenesis of autoimmune diseases not only through auto-antibody secretion but also via cytokine production. Therapeutic depletion of B-cells influences the functions and maintenance of various T-cell subsets. The mechanisms governing the functional heterogeneity of B-cell subsets as cytokine-producing cells are poorly understood. B-cells can differentiate into two functionally polarized effectors, one (B-effector-1-cells) producing a Th-1-like cytokine pattern and the other (Be2) producing a Th-2-like pattern. IL-12 and IFN-γ play a key role in Be1 polarization, but the initial trigger of Be1 commitment is unclear. Type-I-interferons are produced early in the immune response and prime several processes involved in innate and adaptive responses. Here, we report that IFN-α triggers a signaling cascade in resting human naive B-cells, involving STAT4 and T-bet, two key IFN-γ gene imprinting factors. IFN-α primed naive B-cells for IFN-γ production and increased IFN-γ gene responsiveness to IL-12. IFN-γ continues this polarization by re-inducing T-bet and up-regulating IL-12Rβ2 expression. IFN-α and IFN-γ therefore pave the way for the action of IL-12. These results point to a coordinated action of IFN-α, IFN-γ and IL-12 in Be1 polarization of naive B-cells, and may provide new insights into the mechanisms by which type-I-interferons favor autoimmunity.

## Introduction

B cells produce cytokines in response to a broad array of stimuli, including microbial products, antigens, and T cells [1], [2]. Cytokine-producing B cells have been identified in blood and lymphoid tissues of mice and humans with autoimmune disorders and infections [1], [3], [4]. Cytokines secreted by B cells can modulate the differentiation and functions of several key immune effectors, such as CD4 and CD8 T cells, NK cells and dendritic cells [5]. This could explain the antibody-independent immunoregulatory functions of B cells observed in several experimental models of infection and autoimmunity [1], [3], [4]. B cell depletion by rituximab, a mouse-human chimeric antibody specific for CD20, has been tested in various hematological and non-hematological autoimmune diseases [5], [6]. Interestly, rituximab can induce extended periods of clinical remission from autoimmune disorders without significantly reducing serum autoantibody titers [7]. In parallel to this clinical benefit, rituximab has been reported to modulate the numbers and functions of regulatory T cells and T cell effectors in several autoimmune diseases [5], [6]. This supports the emerging concept that B cells may have a pathogenic action which is independent of their antibody production [1]. The mechanisms that control cytokine production by B cells are therefore drawing increasing attention. B cells can differentiate into two distinct Th-1-like and Th-2-like effector subsets that produce distinct polarizing cytokines such as interferon (IFN)-γ and interleukin (IL)-4, respectively [5], [8], [9], [10], [11]. IFN-γ is a key immunoregulatory cytokine and a hallmark of Th-1 responses. We have previously shown the key role of IL-12 and IFN-γ in the generation of IFN-γ-producing B cells [8]. IL-12 triggers STAT4 activation and IFN-γ production by B cells independently of T-bet, which is not directly induced by IL-12. IFN-γ in turn triggers STAT1 activation, T-bet expression, and also its own expression through an autocrine loop [8]. In this IFN-γ double-wave model, IFN-γ acts downstream of IL-12. However, IFN-γ is a major inducer of IL-12Rβ2 expression, a key component in IL-12 signaling [8], raising the possibility that IFN-γ or another, unidentified IL-12Rβ2-inducing factor may also act upstream of IL-12. The initial source of IFN-γ could be innate immune cells such as NK cells or γδ T cells, or B cells themselves, via a third player released early in the immune response. Type I interferons (IFN-α/β) are induced early in the immune response and provide a priming mechanism that orchestrates several subsequent processes involved in innate and adaptive immune responses [12]. They are also involved in the pathogenesis of several systemic and organ-specific autoimmune diseases [12]. Like IL-12, type I IFNs signal through STAT4 and promote IFN-γ secretion by human T cells and mouse T and NK cells [13], [14], [15], [16], [17], [18]. STAT4 activation by type I interferons is critical for the IFN-γ response to viral infections in mice [16]. Here we examined the effects of IFN-α on Be1 polarization.

## Results and Discussion

### IFN-α induces STAT4 activation and T-bet expression in human B cells

**Figure 1:**
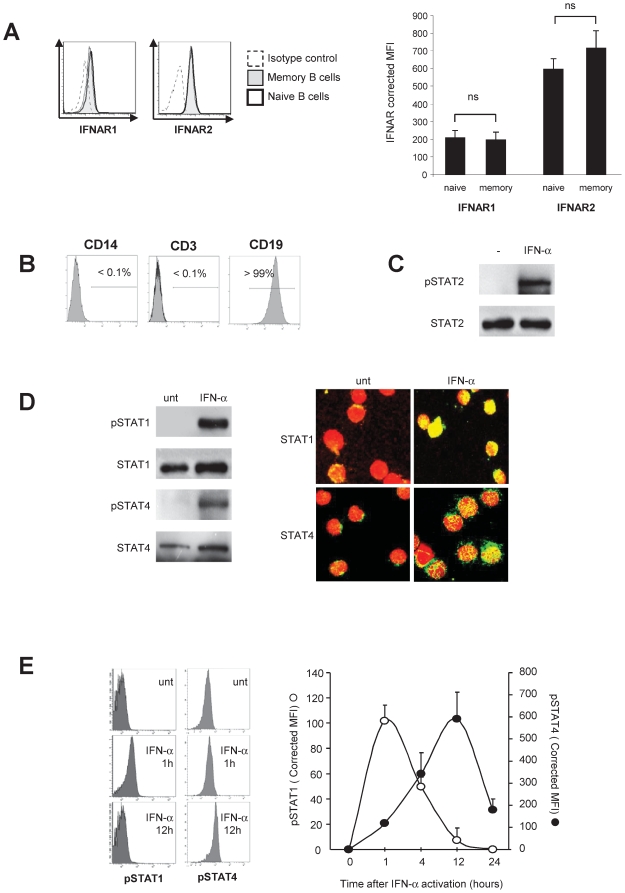
IFN-α induces STAT4 activation in human B cells. In **1A**, IFNAR1 and IFNAR2 expression was analyzed in the CD3^−^CD19^+^CD27^+^ and CD3^−^CD19^+^CD27^−^ lymphocyte gates. A representative staining profile is shown in the left-hand graph. The right-hand graph represents corrected mean fluorescence intensities (MFI) of IFNAR1 and IFNAR2 after substraction of MFI values obtained in isotype control in naive (CD27^−^) and memory (CD27^+^) B cell subsets. The results correspond to the mean ± SEM of the values obtained with cells from 6 healthy donors. **1B** shows the purity of B cell preparations (see methods). In **1C**, purified B cells were activated with IFN-α for 1 h then lysed. Western blotting was performed on whole-cell lysates by using anti-phospho-STAT2, and the membranes were reprobed with anti-STAT2. The data shown in **1C** are representative of 2 independent experiments. **1D, left panel**: B cells were activated for 1 hour with IFN-α. Western blotting was performed on whole-cell lysates by using anti-phospho-STAT1 or anti-phospho-STAT4. The membranes were then reprobed with anti-STAT1 or anti-STAT4. The data shown in **1D**, **left panel** are representative of 2 independent experiments. **1D, right panel**: B cells were activated with IFN-α for 1 hour or left untreated. They were then fixed, permeabilized, and stained with anti-STAT4 or anti-STAT1 (green) plus propidium iodide (nuclear staining, red). Nuclear translocation was examined by confocal microscopy. Yellow spots indicate nuclear STAT. Similar results were obtained in three other experiments. In **1E**, the kinetics of STAT1 and STAT4 phosphorylation was analyzed in B cells by flow cytometry with phospho-STAT-specific antibodies. A representative staining profile is shown in the left-hand graph. The right-hand graph represents corrected mean fluorescence intensities (MFI), after subtraction of MFI values obtained in isotype controls, of phospho-STAT1 and phospho-STAT4 in B cells. The data shown in 1E are the mean ± SEM for cells from 4 healthy donors.

IFN-α induces STAT4 activation and T-bet expression in human B cells. Type I IFNs share a heterodimeric receptor composed of IFNAR1 and IFNAR2 subunits [12]. IFNAR2 appears to serve as the ligand binding chain, but both chains are required for signal transduction [12]. IFNAR1 and IFNAR2 were both expressed at the surface of human resting B cells, with no significant difference in the levels of expression between CD27^−^ naive and CD27^+^ memory B cells (Fig. 1A). We used highly purified human B cells to examine IFN-α signalling (Fig. 1B). Western blot analysis showed that IFN-α phosphorylates STAT2 in human B cells (Fig. 1C) This was consistent with what was observed in T and NK cells [14], [19], [20]. In those cells, it has been shown that PSTAT2 may serve as an adaptor for the recruitment and phosphorylation of STAT1 and, possibly, STAT4 [14], [19], [20], although STAT4 phosphorylation has been observed in response to type I IFN in STAT2-deficient NK cells [15]. B cells constitutively expressed STAT1 and STAT4, while no pSTAT1 or pSTAT4 was detected in untreated cells (Fig. 1D). Cell treatment with IFN-α led to tyrosine phosphorylation of STAT1 and STAT4 (Fig. 1D). No significant nuclear localization of STAT1 and STAT4 was observed in untreated cells, while IFN-α treatment induced nuclear translocation of both factors (Fig. 1D). We used flow cytometry to follow the activation kinetics of STAT1 and STAT4 upon IFN-α exposure. We found sequential kinetic patterns (Fig. 1E): STAT1 activation peaked at 1 hour and had returned to baseline by the time STAT4 activation peaked, after approximately 12 hours (Fig. 1E). No significant difference was found between CD27^−^ naive and CD27^+^ memory B cells in terms of STAT1 and STAT4 phosphorylation levels or kinetics (not shown). This pattern of sequential activation might be related to the relative abundance of STAT1 and STAT4 or to the involvement of suppressor of cytokine signaling (SOCS) proteins such as SOCS1, which inhibits STAT1 activation [21].

**Figure 2:**
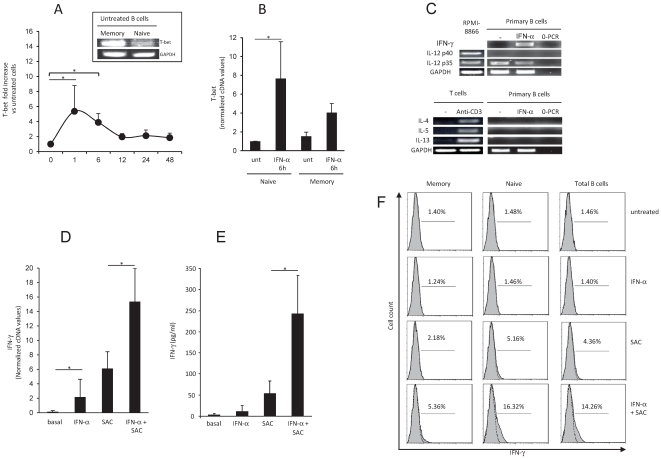
IFN-α induces T-bet expression in B cells and primes B cells for IFN-γ production. In **2A**, T-bet mRNA expression at rest, assessed by RT-PCR, in untreated sorted naive and memory B cell subsets is shown. T-Bet expression was also quantitatively analyzed quantitative RT-PCR in total B cells after IFN-α treatment. Results are expressed as fold increases versus untreated total B cells. In **2B**, T-bet mRNA expression was analyzed in sorted naive and memory B cell subsets after IFN-α treatment for 6 hours. **2C**, B cells were treated for 24 h with IFN-α, and cDNAs were amplified with primers for IFN-γ, IL-12 p40, IL-12 p35, IL-4, IL-5, IL-13 and GAPDH. Positive controls consisted of the EBV-transformed B cell line RPMI-8866 (which constitutively expresses IL-12p35 and IL-12p40) and anti-CD3-activated T cells. **2D, 2E**: B cells pretreated with IFN-α for 18 hours were treated with SAC for 24 hours (for mRNA quantification, 2D) or 48 hours (for ELISA, 2E). The corresponding cDNA and supernatants were assayed for IFN-γ expression by quantitative PCR and ELISA, respectively. **Fig. 2F** shows IFN-γ expression in naive and memory B cell subsets after IFN-α pretreatment and SAC activation for 12 hours, as determined with flow cytometry. Dashed lines correspond to isotype control. Data are representative of 4 different donors (**2C, 2F**) or are mean± SEM of 6 to 7 different donors (**2A, 2B, 2D, 2E**). Statistically significant differences are indicated by an asterisk.

T-bet is a key transcription factor for both Th-1 and Be1 differentiation [8], [9], and its expression is under the control of STAT1 [22]. Both naive and memory B cells constitutively expressed T-bet (Fig. 2A, 2B). As IFN-α activated STAT1 in B cells, we examined the effect of IFN-α on T-bet expression. We found that IFN-α induced a rapid increase in T-bet expression, with a maximum 1 h after activation and a subsequent rapid fall (Fig. 2A). The kinetics of T-bet expression by B cells on IFN-α exposure matched the kinetics of STAT1 activation (Fig. 1E, 2A). By contrast to STAT1 and STAT4, the T-bet-inducing effect of IFN-α mainly concerned the naive subset (Fig. 2B).

### IFN-α may pave the way for Th-1-like B cell differentiation

STAT4 and T-bet are two IFN-γ gene imprinting factors crucial for Th-1-type differentiation [23], [24], [25]. As shown in Fig. 2C and 2D, treatment of human resting B cells with IFN-α led to detectable IFN-γ mRNA expression. By contrast, IFN-α had no effect on the mRNA expression of IL-12 p40 or of Th-2-type cytokines (Fig. 2C). This was consistent with the lack of effect of IFN-α on the activation of STAT6 (not shown), a key regulator of IL-4 and IL-13 gene expression. IFN-α induced barely detectable IFN-γ secretion by resting B cells (Fig. 2E). The mitogenic and polyclonal B cell activator *Staphylococcus aureus* Cowan strain (SAC) induces B cell activation via BCR and Toll-like-receptor 2 stimulation [26]. B cell treatment with IFN-α prior to activation with SAC triggered higher mRNA expression and IFN-γ production (Fig. 2D, 2E). Flow cytometry showed that the naive CD27^−^ B cell subset was mainly responsible for this IFN-γ expression (Fig. 2F). These results suggested that IFN-α may prime naive B cells to produce IFN-γ. Activation of B cells with IFN-α and anti-CD40, anti-IgG, or with a combination of anti-Ig and anti-CD40 did not lead to significant IFN-γ production (not shown), suggesting that the IFN-γ-inducing effect of IFN-α requires additional signals such as TLR signaling, in keeping with previous observations suggesting that TLR signaling is required for optimal naive B cell activation [27], [28]. However isolated TLR2 activation had no effect on IFN-γ expression [29].

**Figure 3:**
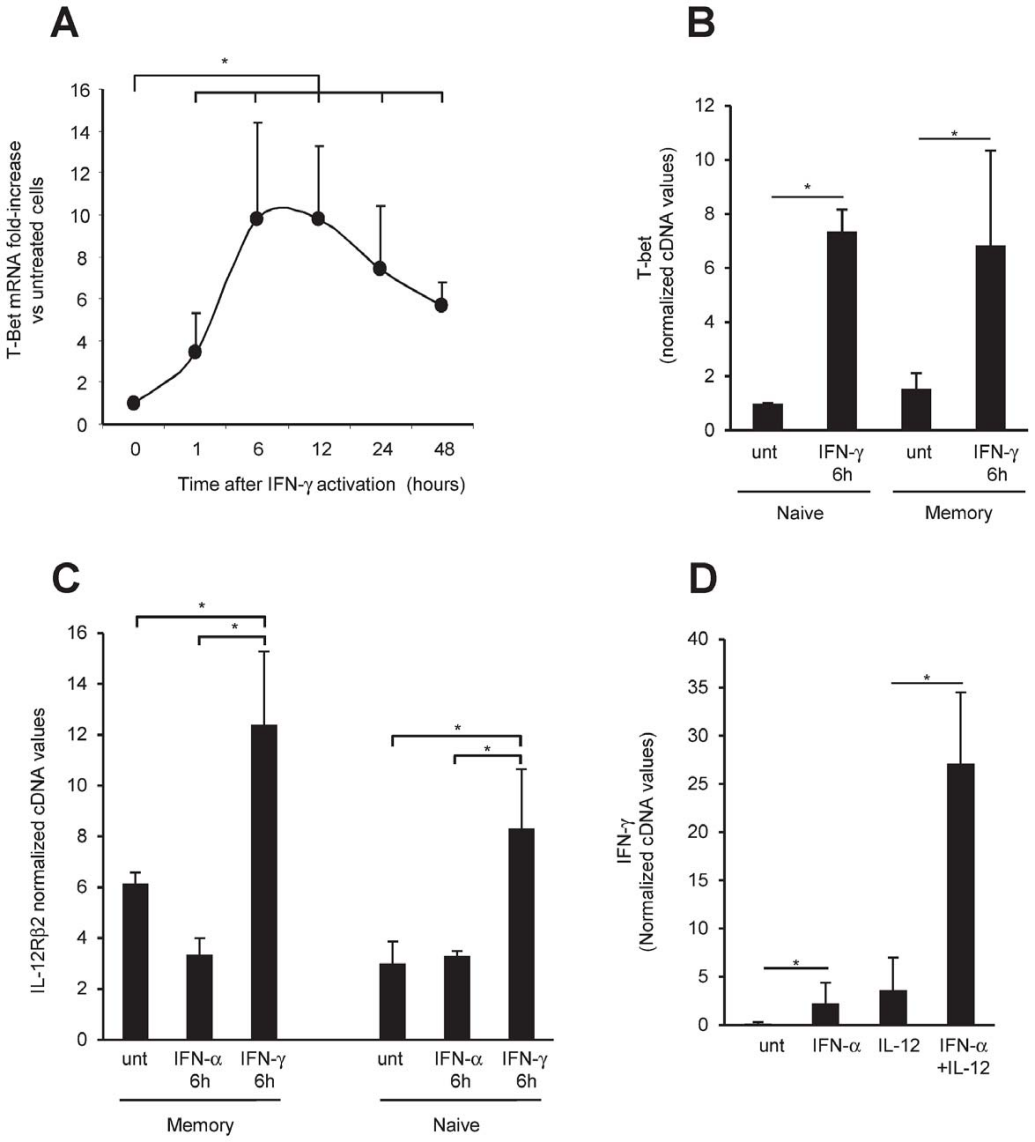
IFN-α increased IFN-γ gene responsiveness to IL-12. 3A, B cells were treated for various times with IFN-γ, and T-bet mRNA was quantified by RT-PCR. The results are expressed as -fold increases versus untreated cells. **3B**: T-bet mRNA -fold increases are shown for the naive and memory subsets at the 6-hour time point. **3C**: Purified naive and memory B cells were activated for 6 hours with IFN-α or IFN-γ. IL-12Rβ2 mRNA expression was analyzed by quantitative PCR. **3D**: B cells were pretreated with IFN-α for 18 h and treated with IL-12 for 24 h, then IFN-γ and GAPDH cDNAs were quantified by PCR. The results are mean ± SEM of values obtained with cells from 4 (**3B**, **3C**) or 6 (**3A**, **3D**) donors. Statistically significant differences are indicated by asterisks.

We have previously reported that IFN-γ induces T-bet expression in B cells [8]. Interestingly, the T-bet-inducing effect of IFN-γ observed here appeared to last longer than that of IFN-α (Fig. 3A) and affected naive and memory subsets to similar extents (Fig. 3B). Another cytokine critical for Be1 and Th-1 commitment is IL-12, and a key step in Th-1 differentiation is the induction of IL-12Rβ2, which is required for effective IL-12 signaling. IFN-γ increased IL-12Rβ2 expression in both naive and memory B cells (Fig. 3C), while IFN-α had no clear effect on IL-12Rβ2 expression, (Fig. 3C). This suggests that IL-12 may mainly act downstream of IFN-α-induced IFN-γ in B cells. This is also in line with the observations that IFN-γ may prime IL-12 production by phagocytic cells [30], [31], [32]. Next, we pre-treated resting B cells with IFN-α, this induced STAT4 activation and T-bet expression as shown above but led only to barely detectable IFN-γ secretion, in the absence of BCR and Toll-like-receptor 2 stimulation (Fig.2E). Despite the lack of significant IFN-γ secretion and IL-12Rβ2 upregulation, IFN-α enhanced IFN-γ expression in response to IL-12 (Fig. 3D). This suggested that the effects of IFN-α on IFN-γ gene synergize with those of a subsequent IL-12 signaling. Therefore, in addition to allow an initial burst of IFN-γ following appropriate B cell antigenic activation, IFN-α may possibly induce epigenetic modifications in the IFN-γ gene that increase responsiveness to IL-12 (Fig. 3D).

### Concluding Remarks

Together, our results identify IFN-α as an initial trigger of Be1 commitment and point to sequential Be1 phenotype imprinting of naive cells by IFN-α, IFN-γ and IL-12, via the action of STAT4 and T-bet. IFN-α triggers IFN-γ production, which in turn promotes IL-12 production by surrounding dendritic cells. IFN-α and IFN-γ enhance B cell responsiveness to IL-12, by increasing IFN-γ gene responsiveness to IL-12 signaling and inducing the β2 component of IL-12R, respectively. The cascade of events triggered by the initial IFN-α signal may therefore prepare the ground for the action of IL-12, which, through sustained effects on STAT4 activation, IFN-γ and T-bet expression may imprint a Th-1-like phenotype on B cells. IL-12 also increases IL-12Rβ2 expression [8], [33].

IFN-α is widely used in the treatment of viral hepatitis, owing to its role in antiviral defenses. However, accumulating evidence also points to a role of IFN-α in the pathogenesis of various hematological, solid organ and systemic autoimmune diseases [12]. The Be1-promoting effect of IFN-α along with the resulting regulation of T cell responses through cross-regulation of Be1/Be2 and Th-1/Th-2 subsets, could be a key factor in the beneficial and deleterious effects of IFN-α.

## Materials and Methods

### Ethics Statement

Human lymphocytes were isolated from anonymous buffy coats obtained from healthy blood donors and provided by the Etablissement Français du Sang (EFS), Hopital S^t^ Louis, Paris, France, in the setting of an agreement signed between the EFS, Hopital S^t^ Louis and INSERM. Informed consents were obtained from the donors by the EFS, Hopital S^t^ Louis. The Bicêtre hospital local ethics committee waived the need for study approval.

### Cells

Highly purified B and T cells were obtained from peripheral blood mononuclear cells (PBMC) following CD19 and CD4 selection in a magnetic separation system, as recommended by the manufacturer (Miltenyi Biotech). B-cell purity was determined by flow cytometry with CD3, CD14, CD16, and CD22 staining (Becton Dickinson). The Epstein-Barr virus-positive (EBV^+^) B-cell line RPMI-8866 was obtained from the European Cell Culture Collection (ECACC). B cell purity was assessed by flow cytometry after CD3, CD14, CD16 and CD22 staining (Becton Dickinson) and always exceeded 98%.

### Flow cytometry

For pSTAT4 and pSTAT1 staining, purified B cells were fixed and permeabilized in cold methanol, then washed and stained for intracellular pSTAT1 or pSTAT4 (BD Biosciences). IFNAR1 and 2 surface expression was examined by using PE-labeled anti-IFNAR2 and FITC-labeled anti-IFNAR1 (R&D systems).

### RT-PCR and quantitative RT-PCR

Purified B cells (2×10^5^/0.2 ml/well) were cultured in 96-well plates in RPMI 1640 medium (Gibco) supplemented with 10% FCS (Biochrom). B cells were activated for up to 72 h with recombinant human IFN-α2b (2000 U/ml) (Schering-Plough S.A.), *Staphylococcus aureus* Cowan I (SAC, 1∶10 000 v/v) (Pansorbin, Calbiochem) or a combination of IFN-α2b and SAC. RNA extraction, reverse transcription and PCR amplification were performed as previously described [8]. The IFN-γ, IL-12p40, IL-12p35, IL-4, IL-5, IL-13 and GAPDH primers and the T-bet primers and probes used here are described elsewhere [8].

T-bet, IFN-γ, IL-12Rβ2, and GAPDH cDNA levels were determined by using Light Cycler-based kinetic quantitative PCR (Roche Diagnostics), as previously reported [8]. To correct for variations in RNA recovery and in the reverse transcription yield, the amounts of T-bet and IFN-γ cDNA were divided by the amount of GAPDH cDNA [8]. T-bet results were expressed as -fold differences in normalized values relative to untreated control cells [8].

### Measurement of STATs

96-well plates were coated for 1 hour at 37°C with poly-L-ornithine and washed 3 times. Thirty thousand purified B cells per well were cultured overnight, then activated with IFN-α2b (2000 U/ml) for various times. The cells were fixed and total STAT4 and total STAT1 were quantified with Elisa methods, as recommended by the manufacturers (STAT4 ELISA, R&D Systems; STAT1 ELISA, Ray-Biotech).

### Confocal microscopy

For confocal microscopic analysis of STAT4 and STAT1 expression, cells were treated with IFN-α2b (2000 U/ml) for 1 hour at 37°C, washed, fixed and permeabilized, then incubated with 10 µg/ml rabbit polyclonal IgG against human STAT4 or STAT1 (Santa Cruz). Indirect immunofluorescence analysis was performed by incubating cells with an Alexa Fluor 488-labeled goat anti-rabbit IgG antibody (10 µg/ml, Molecular Probes). In control experiments the specific antibodies were replaced by a rabbit IgG isotype control (Santa Cruz). Nuclear staining was performed with propidium iodide (250 ng/ml, Sigma Aldrich). After staining, the cells were washed with PBS, centrifuged in a Cytospin 3 (Shandon) and analyzed by laser scanning confocal microscopy with the Leica TCS Confocal System.

### Western blot

Purified B cells were incubated in RPMI 1640 medium containing 10% FCS and treated with IFN-α2b (2000 U/ml) for 1 h at 37°C. They were then washed with PBS containing 50 µM sodium orthovanadate and resuspended in lysis buffer with a protease inhibitor cocktail (Roche Diagnostics). Proteins were fractionated by 8% sodium dodecyl sulfate-polyacrylamide gel electrophoresis (SDS-PAGE) in reducing conditions, then transferred to polyvinylidene difluoride filters (PVDF, Boehringer Mannheim) and probed with rabbit polyclonal anti-pSTAT4 IgG (Zymed), rabbit polyclonal anti-pSTAT2 IgG (Upstate Biotechnology) or rabbit polyclonal anti-pSTAT1 IgG (New England Biolabs), followed by the secondary antibody (horseradish peroxidase (HRP)-conjugated goat anti-mouse or anti-rabbit IgG (Jackson Immunoresearch). PVDF blots were developed with SuperSignal WestPico kits (Pierce). The membranes were then washed and reprobed with anti-STAT4 (Santa Cruz Biotechnology), anti-STAT2 or anti-STAT1 (rabbit polyclonal IgG, Upstate Biotechnology).

### IFN-γ production

Purified B cells (1×10^6^ per well) were cultured in 48-well plates in 500 µl of 10% FCS/RPMI 1640 medium. They were pretreated with recombinant IFN-α2b (2000 U/ml) for 18 h then activated with SAC (1∶10 000 v/v) for 48 h. IFN-γ was assayed in the supernatants (human IFN-γ ELISA, R&D).

### Statistical Analysis

Wilcoxon paired t-test was used with Bonferroni correction.
